# Fracture Toughness and Failure Mechanisms of Glass-Reinforced Plastics Based on an Epoxy Matrix Modified with Polysulfone and an Active Diluent

**DOI:** 10.3390/polym18080991

**Published:** 2026-04-19

**Authors:** Tuyara V. Petrova, Ilya V. Tretyakov, Olga V. Alexeeva, Andrey Yu. Sergeev, Roman A. Korokhin, Vitaliy I. Solodilov, Gleb Yu. Yurkov, Alexander Al. Berlin

**Affiliations:** 1 N.N. Semenov Federal Research Center of Chemical Physics, Russian Academy of Sciences, 119991 Moscow, Russia; tretiakovi.v@yandex.ru (I.V.T.); sergeevandrey89@gmail.com (A.Y.S.); korohinra@gmail.com (R.A.K.); vital-yo@yandex.ru (V.I.S.); berlin@chph.ras.ru (A.A.B.); 2 N.M. Emanuel Institute of Biochemical Physics, Russian Academy of Sciences, 119334 Moscow, Russia; alexol@yandex.ru

**Keywords:** fracture toughness, polysulfone, active diluent, epoxy matrix, glass-fiber-reinforced plastics

## Abstract

The crack resistance of unidirectional fiberglass-reinforced plastics based on an epoxy matrix modified with polysulfone (PSU) and furfuryl glycidyl ether (FGE) was investigated. The combined addition of PSU/FGE modifiers to the epoxy matrix increases the crack resistance of glass-fiber-reinforced plastics (GFRPs). The effect of increasing the crack resistance of GFRPs varies depending on the modifier ratio. The greatest increase in crack resistance is achieved with a modifier ratio of 1/0.5. For this ratio, the value of $G_{IR}^{CM}$ is 1.18 kJ/m^2^ (for unmodified GFRP, $G_{IR}^{CM}$ = 0.72 kJ/m^2^). With an increase in the FGE concentration in the polysulfone-modified epoxy matrix, the crack resistance of GFRP decreases to a level of ~0.8 kJ/m^2^. The change in the crack resistance of GFRP is associated with the structure of the epoxy matrix containing different PSU/FGE ratios. A study of the fracture surfaces of GFRPs showed that the greatest increase in the crack resistance of composites is achieved with the formation of extended phases enriched with polysulfone in the epoxy matrix. The size of the dispersed phase is about 3 μm. A correlation has been established between the crack resistance of hybrid matrices and GFRPs. With an increase in the matrix crack resistance by 3.1 times (from 0.37 to 1.15 kJ/m^2^), the fracture toughness value of GFRP increased by 1.6 times (from 0.72 to 1.18 kJ/m^2^).

## 1. Introduction

Fiber-reinforced plastics based on epoxy polymers are widely used as structural materials in various fields of engineering [1,2]. In such materials, the main stresses are absorbed by the fibers, which provide the rigidity and strength of the material. However, epoxy matrices are characterized by a high density of the molecular network after curing, which leads to their reduced resistance to the formation and propagation of cracks [3,4,5,6]. During service, microcracks will accumulate in reinforced plastics. The accumulation and propagation of microcracks can be prevented by creating microplastic structures in the polymer matrix. Microcracks that arise in such a matrix (a hybrid polymer matrix) will initiate and propagate at high energies, which will ultimately increase the fracture toughness of the materials. A hybrid polymer matrix is a matrix structure containing phases of different polymeric nature: a cross-linked thermosetting polyepoxide and a rigid-chain thermoplastic polymer. In the vast majority of cases, the creation of hybrid polymer matrices involves the phase separation of epoxy–polymer mixtures during curing. The second polymer used is a modifier, such as rigid-chain thermoplastics. Depending on the curing conditions (temperature, modifier concentration, and curing rate), heterogeneous phases of varying structure are formed. The type of supramolecular structure largely determines the resistance of the hybrid polymer matrix to crack propagation [7,8,9,10,11]. Thus, the phase structures formed in free volume and under conditions of confinement by reinforced fibers in reinforced plastics can differ significantly [12,13].

The greatest effects in increasing the fracture toughness of epoxy matrices are observed when using rigid-chain thermoplastics such as polysulfones [14,15,16], polyetheretherketones [17,18], and polyetherimides [19,20,21]. In the epoxy oligomer–thermoplast polymer mixture, phase separation occurs during the curing process. As a result of the formation of a heterogeneous structure in the matrix, the fracture toughness increases due to the dissipation of crack growth energy in the more plastic phase enriched with thermoplastic. However, this modification significantly increases the viscosity of epoxy–polymer mixtures, which imposes significant technological limitations on the production of reinforced plastics [22,23,24]. Viscosity is typically adjusted by adding organic or active diluents to the epoxy binder [25,26,27,28]. The use of organic low-boiling solvents can lead to increased defects in the material and a decrease in its strength and crack resistance. Therefore, the use of active diluents for epoxy–thermoplastic mixtures is preferable, as this will prevent the formation of volatile compounds that can impair the structure of the reinforced plastic. However, introducing an additional component into the epoxy–polymer mixture can firstly alter the structure of the hybrid matrix and secondly the entire range of properties of the reinforced plastic.

In a previous study [29], we obtained hybrid epoxy binders containing polysulfone, which increased the dissipative properties of the epoxy matrix, and furfuryl glycidyl ether as an active diluent. The use of furfuryl glycidyl ether to reduce the viscosity of the mixture improved the processing properties of high-viscosity binders. It was shown that the formation of a continuous thermoplastic-rich phase significantly (from 0.37 to 1.15 kJ/m^2^ for the amine curing agent and from 0.17 to 0.9 kJ/m^2^ for the anhydride curing agent) increased the crack resistance of hybrid matrices. In this case, the active diluent shifts the region of formation of such a structure to the region with a high concentration of thermoplastic.

In fiber-reinforced plastics, the free volume of the matrix is limited by the fibers, which affects the heterogeneous structure of the matrix. In [13,30], we showed that the matrix fracture toughness and the fracture toughness of epoxy plastics reinforced with glass, carbon, or aramid fibers differ significantly. Moreover, epoxy matrices modified with thermoplastics (polysulfone or polyethersulfone) were dual-phase systems.

In this study, we investigated the fracture toughness of unidirectional fiberglass plastics based hybrid matrices obtained from ternary systems of epoxy oligomer–polysulfone–furfuryl glycidyl ether. In contrast to [29], the formation of the phase structure of hybrid (ternary) matrices occurred in a volume constrained by glass fibers, i.e., in the reinforcing structure of fiberglass plastic. Analysis of the fracture surface of GFRP and the matrix structures involved in the fracture process will allow us to expand our understanding of the nature of crack initiation and propagation not only in matrices but also at the composite material level. The data obtained from these studies will allow us to predict the resistance of fiberglass composites based on hybrid matrices to crack propagation.

## 2. Materials and Methods

### 2.1. Materials

The epoxy oligomer CHS EPOXY 520 (EO, Spolchemie, Ustin nad Labem, Czech Republic), polysulfone PSK-1 (PSU, JSC NIIPM, Moscow, Russia) with a molecular weight of 35,000, active diluent furfurylglycidyl ether (FGE, LLC DOROS, Yaroslavl, Russia), and curing agent triethanolamine titanate were used in this study (TEAT, JSC CHIMEX Limited, St. Petersburg, Russia), with RVMPN 10-400-80 glass roving (NPO Stekloplastic, Moscow, Russia).

### 2.2. Preparation of Binders

To obtain the polymer mixture, PSU was dissolved in EO at a temperature of 100–120 °C. The active diluent FGE was added to the resulting mixture at a temperature of 60–80 °C. Then, the curing agent TEAT was added.

The component content in the binder is shown in Table 1.

### 2.3. Preparation of GFRP

**Figure 1:**
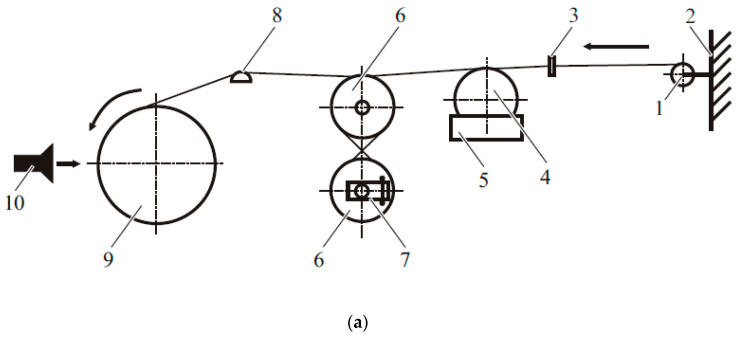

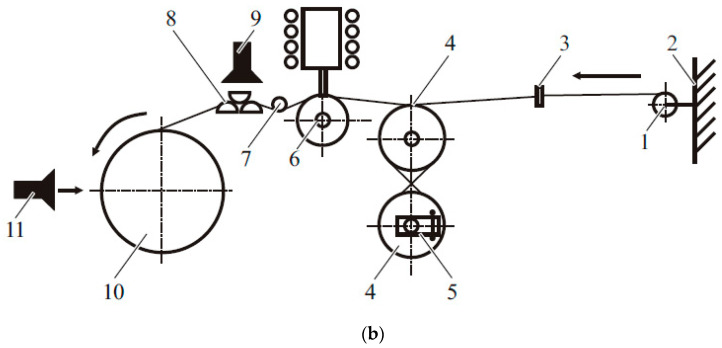
Scheme of an impregnation-tensioning path for winding laboratory specimens of reinforced plastics with low-viscosity (**a**) and high-viscosity (**b**) binders: (**a**) 1—bobbin; 2—bobbin holder; 3—guiding ring; 4—impregnation roller; 5—heated tray; 6—tension drums; 7—braking device; 8—distributor; 9—mandrel; 10—air heater. (**b**) 1—bobbin; 2—bobbin holder; 3—guiding ring; 4—tension drums; 5—heated tray; 6—capillary viscometer; 7—roller; 8—impregnating device; 9 and 11—air heater; 10—mandrel. The sample preparation technology is described in detail in [31].

Unidirectional fiberglass-reinforced plastics (GFRPs) were produced using the winding method on cylindrical mandrels as described in [31]. The study describes two technological schemes; the first is typical for low-viscosity binders, and the second is for high-viscosity binders (Figure 1). According to the technological parameters obtained in [32] for high-viscosity binders for processing compositions, winding was carried out at a temperature of 80–100 °C and a viscosity of the PSU/FGE system in a ratio of 0.75/1, which made it possible to carry out winding using the classical processing method, as well as an unmodified binder. After winding, the mandrels were removed from the winding machine and cured in a thermal cabinet for 8 h at 160 °C.

After curing, the wound rings were cut into segments with a length of 110–115 mm, a width of 10 mm, and a thickness of 2 mm. The radius of curvature of each segment was 75 mm. The volume fraction of fibers in the obtained composites was 71–75 vol.%. The void content was 1–2 vol.% The parameters of GFRPs were determined according to ISO 1172:2023 [33]. 

### 2.4. Characterization and Measurement

The energy of interlayer fracture of GFRP $G_{IR}^{CM}$ was determined by splitting a double-cantilever beam using the “angle method” [34].

The tests were carried out on a Zwick/Roell Z100 universal testing machine (ZwickRoell GmbH & Co., Ulm, Germany). The loading rate was 50 mm/min.

An initial crack of ~10 mm was created at the winding stage in the middle layer of the sample using a Teflon film. Then, 1.5 mm deep cuts were made at the ends of each segment, 2 mm from the specimen edge, to secure steel wire loops through which the external load was applied. Each batch consisted of three samples.

The sample was loaded until the crack grew by approximately 10 mm; then, the load was removed, and photographs were taken to determine the bending angles of the consoles, α_1_ and α_2_. The loading cycle was then repeated at least five times. During the test, the dependence of the force *F* on the crack opening magnitude *D* was recorded.

The energy of interlayer destruction $G_{IR}^{CM}$ was calculated using the following formula [34]:

(1) $$G_{IR}^{CM}=\frac{F_{c}}{w}\left(sin\alpha_{1}+sin\alpha_{2}\right),$$

where *w* is the width of the sample.

**Figure 2:**
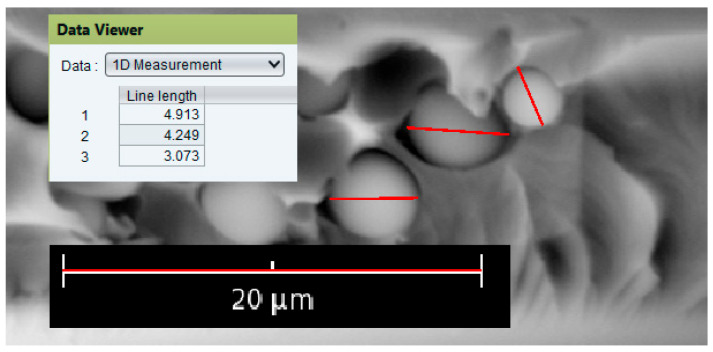
Particle size measurement using JMicroVision software.

The fracture surface morphology of fiberglass composites after cracking was examined using scanning electron microscopy (SEM). A sample was taken from the crack propagation region. The studies were performed on a Phenom ProX SEM (Thermo Fisher Scientific, Waltham, MA, USA). The resulting micrographs were analyzed using JMicroVision v. 1.3.4 software, and the sizes of the dispersed phases enriched with polyepoxide were measured. To measure particle size in the software, the size was calibrated using a micrograph scale bar (Figure 2). After calibration, the particle diameter was marked using the lines and displayed in a separate window. The program then generated a size distribution graph. Approximately 100 dispersed particles were measured for each composition.

## 3. Results and Discussion

### 3.1. Fracture Toughness of GFRP

**Figure 3:**
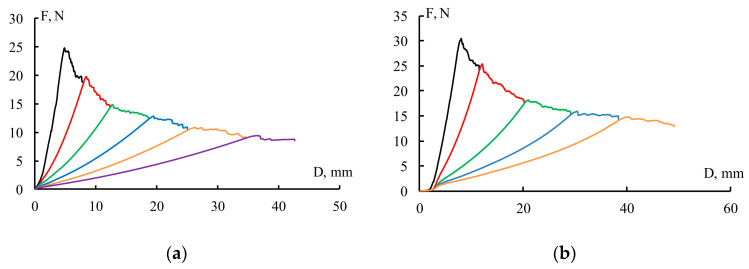
Load diagrams of unidirectional GFRP based on epoxy matrix containing PSU/FGE: (**a**) reference; (**b**) 1/0.5.

Figure 3 shows the *F-D* typical load diagrams for fiberglass composites based on unmodified and modified matrices. The typical load diagrams shown in Figure 3 were selected based on the minimum crack resistance corresponding to unmodified GFRP (Figure 3a) and the maximum crack resistance of GFRP (Figure 3b), achieved by introducing PSU/FGE modifiers into the epoxy matrix in a ratio of 1/0.5. Therefore, the load diagrams correspond to the limits of the range of the materials studied. For both types of samples, crack growth is observed upon reaching the maximum load, after which the load decreases. The load fluctuations are slight, indicating smooth crack propagation. After the crack reaches a given length, the sample is unloaded. The load is then reapplied, the crack grows, and the sample is unloaded. This cycle is repeated several times. Modifying the epoxy matrix with polysulfone and an active diluent does not change the appearance of the load diagrams. However, the level of load required for crack propagation differs from that of fiberglass based on an unmodified matrix. It should be noted that for GFRP based on epoxy matrices modified with other PSU/FGE ratios, the type and nature of the load diagrams do not change.

**Figure 4:**
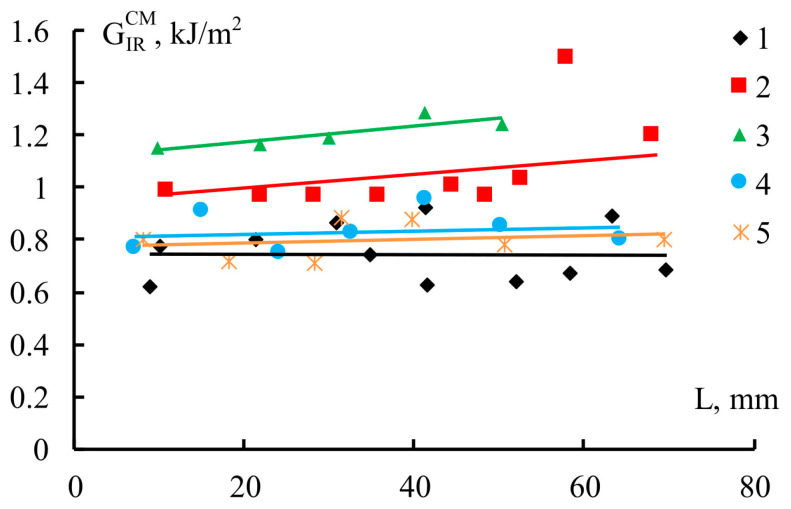
Fracture toughness, $G_{IR}^{CM}$, versus crack length, *L*, for unidirectional GFRP based on epoxy matrix modified with PSU/FGE: 1—reference; 2—1/0; 3—1/0.5; 4—1/1; 5—0.75/1.

Based on the obtained loading diagrams, the values of the specific fracture toughness of fiberglass-based composites containing PSU and FGE were calculated. Figure 4 shows the fracture toughness, $G_{IR}^{CM}$, of fiberglass-reinforced plastics versus crack length, *L.*

The fracture toughness of reinforced plastics based on the studied matrices is practically independent of crack length. The independence of the $G_{IR}^{CM}$ values from the crack length indicates that the reinforced plastic delaminates without forming strands [35]; i.e., the surfaces of the growing crack are not connected by reinforcement fibers, which can contribute to the energy of delamination of the reinforced plastic. Modifying fiber-reinforced plastics with varying amounts of modifiers results in different levels of crack resistance. For unmodified GFRP (Figure 4), the typical $G_{IR}^{CM}$ value is approximately 0.75 kJ/m^2^. The introduction of PSU and FGE modifiers into the GFRP epoxy matrix increases their fracture toughness to values exceeding 0.8 kJ/m^2^.

The average fracture toughness of unidirectional GFRPs based on TEAT-cured matrices with different PSU and FGE contents is shown in Table 2.

The introduction of PSU into the epoxy matrix increases the $G_{IR}^{CM}$ value of fiberglass by 56% (from 0.72 to 1.12 kJ/m^2^). Modification of the epoxy–polysulfone matrix with an active diluent shows that the fracture toughness of fiberglass based on a matrix with a PSU/FGE content of 1/0.5 remains at the level of the epoxy–polysulfone matrix ($G_{IR}^{CM}$ = 1.18 kJ/m^2^). Adding more FGE to the epoxy–polysulfone matrix reduces the crack resistance of reinforced plastics. For a fiberglass-reinforced plastic based on a matrix with a PSU/FGE ratio of 1/1, the $G_{IR}^{CM}$ value is 0.83 kJ/m^2^. It should be noted that the obtained fracture toughness values of GFRP based on a matrix with a PSU/FGE content in a ratio of 1/1 are significantly higher (by 15%) than the fracture toughness of unmodified GFRP.

The introduction of PSU/FGE into the epoxy matrix in a ratio of 0.75/1 does not reduce the crack resistance of GFRP compared to unmodified materials.

### 3.2. Structural and Morphological Study

**Figure 5:**
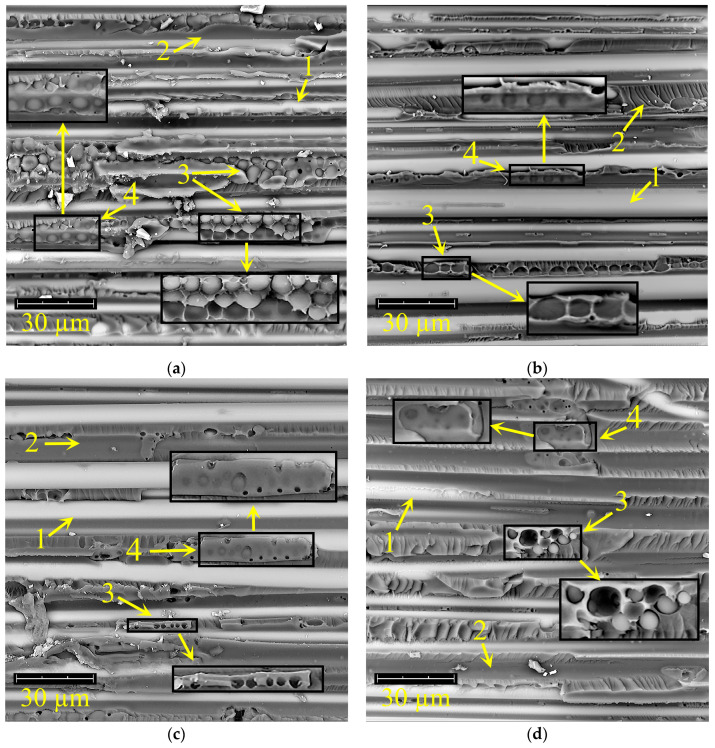
Micrographs of the fracture surface of fiberglass based on a modified matrix containing PSU/FGE: (**a**) 1/0; (**b**) 1/0.5; (**c**) 1/1; (**d**) 0.75/1. 1—matrix; 2—reinforcing fiber; 3—inverted phase; 4—undamaged areas of the inverted phase.

Figure 5 shows micrographs of the fracture surfaces of fiberglass composites. The fracture of reinforced plastics occurs predominantly at the interface between the matrix (2) and the reinforcement fiber (1). Nevertheless, there are areas of reinforcing fibers where some parts of the polymer matrix remain. Regions with an inverted phase (3) also participate in the propagation of the crack.

Inverted-phase regions (3) are observed in the GFRP matrix with polysulfone content (Figure 5a). In this case, the matrix is enriched with thermoplastic, and the dispersed phase is EO. The size of the EO-enriched dispersed phase ranges from 0.8 to 6.8 µm. During composite delamination, the growing crack does not always propagate through the inverted phase.

There are areas where there is delamination between the epoxy matrix (2) and the reinforcement fiber (1), and the matrix shows undamaged areas of the inverted phase (4). It should be noted that the inverted phase is in contact with the reinforcing fibers. The fracture toughness of GFRP based on the polysulfone-containing system is 1.12 kJ/m^2^ (Table 2).

When an active diluent is added to the epoxy–polysulfone matrix in a 1/0.5 ratio (PSU/FGE), the size of the dispersed epoxy phase in the inverted structure remains almost unchanged at 0.7–6.4 μm (Figure 5b). In the polymer matrix (2) of GFRP, the presence of a finely dispersed phase enriched with polysulfone, up to 1 μm in size, was detected (Figure 5b). The failure of GFRP based on a system with a PSU/FGE content of 1/0.5 occurs through the same mechanism as for the epoxy–polysulfone system. Fracture toughness values change little (1.18 kJ/m^2^) (Table 2).

With increasing concentration of the active diluent, i.e., for GFRP based on a matrix with a PSU/FGE content in a ratio of 1/1, a similar phase structure is observed (Figure 5c). This leads to an increase in the amount of EO-rich dispersed phase with a larger particle size (1.3–7.4 µm). Furthermore, the amount and size (up to 2 µm) of the finely dispersed phase in the GFRP matrix (2) increases. The failure of fiber-reinforced plastics occurs through the mechanism described above. Reducing the amount of destroyed inverted phases (3) leads to a decrease in its contribution to crack propagation. Thus, crack growth energy is dissipated less efficiently. The fracture toughness values for GFRP based on a matrix containing a 1/1 PSU/FGE ratio decreased to 0.83 kJ/m^2^ (Table 2).

For GFRP, the fracture toughness value with a polysulfone content of 0.75/1 (PSU/FGE) differs little from the fracture toughness for GFRP with a PSU/FGE content of 1/1 and is 0.82 kJ/m^2^ (Table 2). The number of regions with an inverted structure decreases compared to systems containing a higher PSU content (Figure 5d). The size of EO-rich phases in the inverted structure ranges from 1.8 to 5.8 μm. There are also fine-grained phases enriched with PSU, with sizes of up to 2 μm.

**Figure 6:**
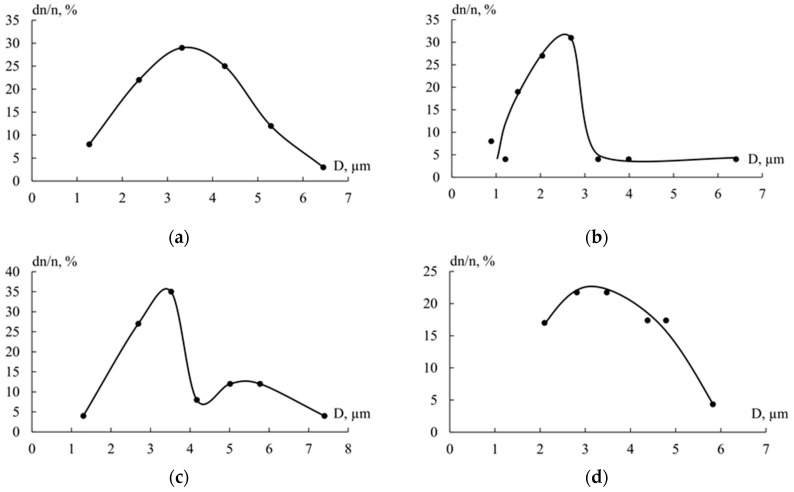
Distribution of the dispersed epoxy phase by size in fiberglass-reinforced plastics based on a modified matrix containing PSU/FGE: (**a**) 1/0; (**b**) 1/0.5; (**c**) 1/1; (**d**) 0.75/1.

The size and size distribution of the dispersed phases in the polymer matrix are shown in Figure 6. From the obtained curves, it follows that for GFRP based on an epoxy–polysulfone matrix (Figure 6a) and a matrix containing PSU/FGE in a ratio of 0.75/1 (Figure 6d), the distribution of particles enriched with polyepoxide is characterized by a distribution close to normal. For the epoxy–polysulfone matrix, the particle size ranges from 0.8 to 6.8 μm, while for 0.75/1 (PSU/FGE) it ranges from 1.8 to 5.8 μm. The most common phase size for these two matrices is approximately 3 μm. For GFRP with a composition of 1/0.5 (PSU/FGE) (Figure 6b), a significant deviation of the polyepoxide-rich phase size from the normal distribution law is observed. It can be seen that the majority of particles range in size from 0.7 to 3 µm, with a small number of particles ranging from 3 to 6.4 µm. The average diameter of the most common EO-rich particles was 2.69 µm. For GFRP based on a 1/1 matrix (PSU/FGE) (Figure 6c), a bimodal distribution of polyepoxide-rich particles is observed. The first group of particles ranges in size from 1.3 μm to 4 μm, while the second group ranges from 4 μm to 7.4 μm. This particle distribution pattern is typically associated with secondary phase separation. During the initial phase separation, large phases enriched in polyepoxide form and grow. With further curing, and as a result of changes in interdiffusion coefficients (disruption of phase equilibrium), a finer polyepoxide phase begins to form and grow. The average size of the most frequently encountered dispersed epoxy phase was 3.52 µm and 5.39 µm. Deviation from the normal distribution of particles in the 1/0.5 composite matrix (PSU/FGE) (Figure 6b) can also be associated with secondary phase decay. The most common sizes of dispersed epoxy particles are given in Table 3.

A significant improvement in fracture toughness was observed in glass-reinforced plastics based on matrices containing the highest amount of dispersed phase enriched with epoxy oligomer, with sizes of 3.32 and 2.69 µm. The content of dispersed phases with such dimensions was about 30%. These parameters correspond to compositions with a PSU/FGE content of 1/0 and 1/0.5. Fracture toughness values at the same level were obtained for fiberglass-based composites with a PSU/FGE content of 1/1 and 0.75/1. Although the size of the most common epoxy dispersed phases was 3.15 μm for fiberglass-based matrices with a PSU/FGE ratio of 0.75/1, their proportion in the fiberglass matrix was small and equal to approximately 23%. For GFRPs based on matrices with a PSU/FGE ratio of 1/1, an increase in the content of EO-rich dispersed phases with a larger size (5.39 μm) was observed.

The change in the phase sizes in the freely polymerizing volume is considered in detail in [29]. It is shown that epoxy matrices modified with PSF and FGE are characterized by a “matrix-dispersion” type structure. The matrix in such a system is enriched with polysulfone, and the dispersed phase is enriched with epoxy oligomer. The addition of FGE to EO + PSF leads to an increase in the size of the dispersed phase enriched with epoxy. This behavior of the system is caused, on the one hand, by a decrease in the viscosity of the system and, as a consequence, an increase in the interdiffusion coefficients of the components. On the other hand, phase decomposition (PD) initiated by the chemical reaction of curing, leading to an expansion of the heterogeneous region of the PD along the concentration and temperature scales, and the intersection of the process isotherm, will occur at lower degrees of conversion. As a result of this and the better mobility of the components in the initial low-viscosity system, the formation of phase structures will begin at higher diffusion coefficients compared to a system without FGE.

Under constrained conditions (when reinforcing fibers, in this case glass, are introduced into the polymerizing system), the nature of phase separation and the laws of phase formation will remain as described above. Only the directions of propagation of the interdiffusion fronts of the binder components will change, which can only affect the localization of phase structures in the space between the reinforcing fibers, as we observed in our study of the surface morphology of fractured fiberglass (see Figure 5).

### 3.3. Dependence of G_IR_ of Reinforced Plastics on the G_IR_ of Matrices

**Figure 7:**
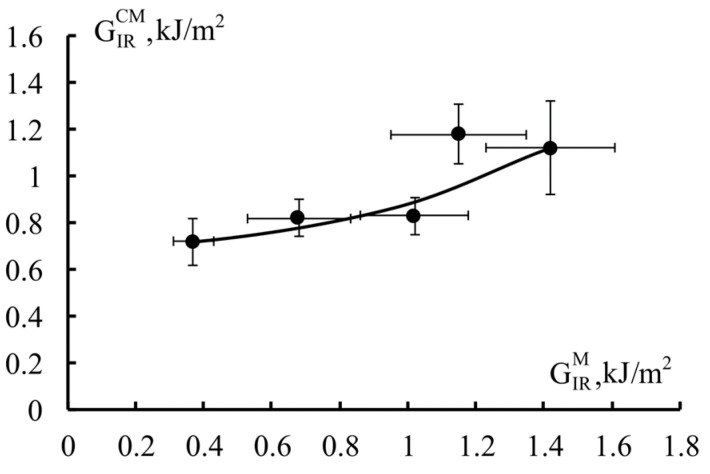
Correlation of fracture toughness of unidirectional glass-reinforced plastics, $G_{IR}^{CM}$, and modified epoxy matrices, $G_{IR}^{M}$.

Based on the obtained values of fracture toughness of fiberglass and fracture toughness of matrices, described previously in [29], the dependence of crack propagation energy in fiberglass on the fracture toughness of matrices was determined (Figure 7). It is evident that with an increase in matrix crack resistance, the fracture toughness of fiberglass increases, and with an increase in matrix crack resistance by 3.1 times (from 0.37 to 1.15 kJ/m^2^), the fracture toughness of fiberglass increases by 1.6 times (from 0.72 to 1.18 kJ/m^2^).

Previously, correlations between the crack resistance of reinforced plastics and matrices for various materials were obtained. In [13], a 4.3-fold (from 0.37 to 1.59 kJ/m^2^) increase in the fracture toughness of an epoxy matrix modified with polyethersulfone (PES) increased the delamination energy of fiberglass by 1.5 times (from 1.01 to 1.51 kJ/m^2^) and carbon fiber by 1.6 times (from 0.35 to 0.66 kJ/m^2^). In [30], a monotonic increase in fracture toughness was observed with an increase in fracture toughness of matrices for glass (GFRP), carbon (CFRP), and organic (OFRP) fiber-reinforced plastics based on an epoxy matrix modified with polysulfone. In this case, a 6-fold increase (from 0.3 to 2.0 kJ/m^2^) in the fracture toughness of the matrices increased the crack resistance of all reinforced plastics by approximately two times (GFRP: from 1.0 to 2.0 kJ/m^2^; CFRP: from 0.25 to 0.5 kJ/m^2^; OFRP: from 1.3 to 2.6 kJ/m^2^).

Thus, for all the reinforced plastics studied, the increase in fracture toughness values depends on the modifier content in the matrix. However, the correlation between the fracture toughness of reinforced plastics and matrices remains the same for all materials. By increasing the fracture toughness of matrices, the interlayer fracture energy of reinforced plastics can be increased. It is important that the formation of the phase structure of the matrix of reinforced plastics occurs in thin layers between the reinforcing fibers. Thus, the resulting phase structure will differ significantly from the phase structure of matrices formed in a free (unconstrained) volume, which is evident from our previously presented works [13,29]. In reinforced plastics, crack propagation will also be influenced by factors such as the presence of reinforcing fibers and the degree of involvement of phase structures in the crack propagation process. Based on the above, it can be concluded that the significant difference between the crack resistance of the matrix and reinforced plastics is primarily associated with all these factors.

## 4. Conclusions

This study investigated the fracture toughness of unidirectional fiberglass composites based on an epoxy matrix modified with polysulfone (PSU) and furfuryl glycidyl ether (FGE). Combined modification of the epoxy matrix with polysulfone and furfuryl glycidyl ether increases the fracture toughness of fiberglass composites. Adding PSU/FGE modifiers in a ratio of 1/0.5 to the epoxy matrix increases the fracture toughness of fiberglass from 0.72 to 1.18 kJ/m^2^. Using other modifier ratios (1/1, 0.75/1, and 1/0) increases the fracture toughness of fiberglass to 0.82–1.12 kJ/m^2^. It should be noted that the fracture toughness of reinforced plastics based on the studied matrices is practically independent of crack length. Furthermore, reinforced plastic delaminates without the formation of strands, which could further contribute to the delamination energy of the reinforcing plastic.

It has been established that the greatest increase in the crack resistance of fiberglass-reinforced plastics is achieved with the formation of extended phases enriched in polysulfone in an epoxy matrix. Depending on the ratio of modifiers in the fiberglass matrix, extended phases enriched in polysulfone are formed during curing, within which a phase enriched in polyepoxide is located. The maximum increase in fracture toughness is observed in the presence of extended polysulfone phases, within which dispersed polyepoxide particles with a diameter of less than 3 μm are distributed. As the size of the dispersed polyepoxide phase increases, a decrease in the fracture toughness of the fiberglass is observed.

A correlation was established between the fracture toughness of hybrid matrices and fiberglass composites. An increase in fiberglass fracture toughness from 0.72 to 1.18 kJ/m^2^ (1.6 times) was achieved with an increase in matrix fracture toughness from 0.37 to 1.15 kJ/m^2^ (3.1 times) [29].

The results obtained in this study show that the enhanced fracture toughness of epoxy–polysulfone–furfuryl glycidyl ether ternary systems is maintained at the composite level when the polymerization volume is limited by fibers. Analysis of the obtained data will make it possible to regulate the crack resistance of fiberglass by modifying epoxy matrices not only with thermoplastics but also by using compatible active diluents in combination with them.

## Figures and Tables

**Table 1:** The component content in the binder.

PSU/FGE Ratio	Component Content			
	EO	PSU	FGE	TEAT
Ref	0.91	-	-	0.09
1/0	0.77	0.15	-	0.08
1/0.5	0.71	0.14	0.07	0.08
1/1	0.66	0.13	0.13	0.08
0.75/1	0.68	0.10	0.14	0.08

**Table 2:** Fracture toughness, $G_{IR}^{CM}$ (kJ/m^2^), of glass-fiber-reinforced plastics.

PSU/FGE Ratio	Reference	1/0	1/0.5	1/1	0.75/1
$G_{IR}^{CM}$, kJ/m^2^	0.72 ± 0.1	1.12 ± 0.2	1.18 ± 0.13	0.83 ± 0.08	0.82 ± 0.08

**Table 3:** The most common size of dispersed particles enriched with polyepoxide.

PSU/FGE Ratio	Reference	1/0	1/0.5	1/1	0.75/1
D, µm	-	3.32 ± 0.31	2.69 ± 0.21	3.52 ± 0.27 5.39 ± 0.49	3.15 ± 0.41

## Data Availability

The original contributions presented in this study are included in the article material. Further inquiries can be directed to the corresponding authors.
